# Supplementary figures and images for: Pan-cancer systematic identification of lncRNAs associated with cancer prognosis
Source: PeerJ. 2020 Mar 24;8:e8797. doi: 10.7717/peerj.8797 (PMC7100599; doi:10.7717/peerj.8797)

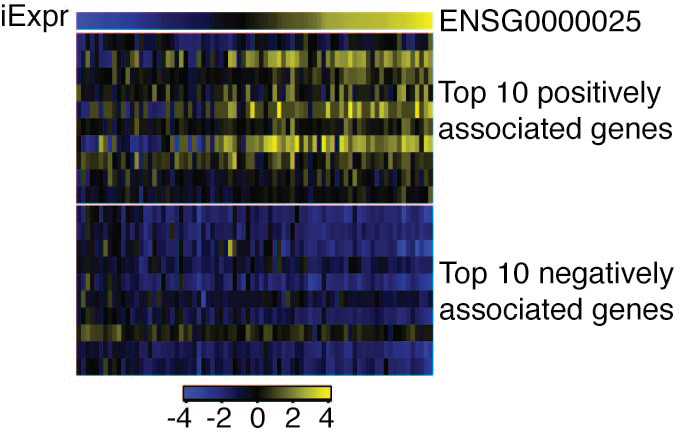

Supplement: Supplemental Information 1 — The expression of twenty genes that have the highest contribution to the inferred expression (iExpr) of lncRNA LINC01684 are depicted. The entire regulon consisted of 820 genes. [file peerj-08-8797-s001.png]

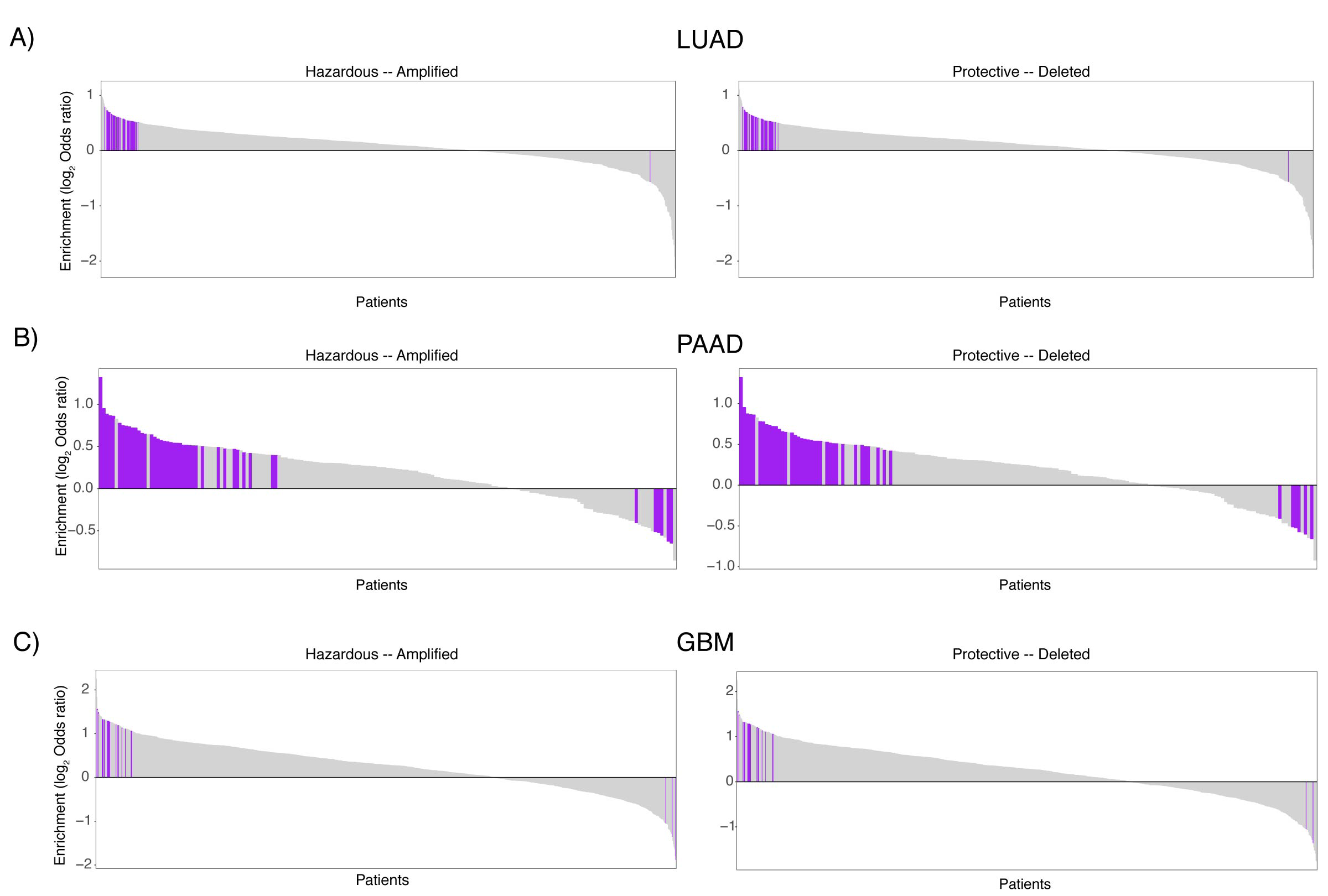

Supplement: Supplemental Information 2 — (A) Enrichment of hazardous and protective lncRNAs in amplified and deleted regions of the genome, respectively, across lung adenocarcinoma patients. (B) Enrichment of hazardous and protective lncRNAs in amplified and deleted regions of the genome, respectively, across pancreatic cancer patients. (C) Enrichment of hazardous and protective lncRNAs in amplified and deleted regions of the genome, respectively, across glioblastoma patients. [file peerj-08-8797-s002.png]
