# Supplementary material for: Pan-cancer systematic identification of lncRNAs associated with cancer prognosis
Source: PeerJ. 2020 Mar 24;8:e8797. doi: 10.7717/peerj.8797 (PMC7100599; doi:10.7717/peerj.8797)
Supplement: Supplemental Information 3 [file peerj-08-8797-s003.pdf]

Supplemental Methods

Pan-cancer systematic identification of lncRNAs associated with cancer prognosis

Matthew H. Ung, Evelien Schaafsma, Daniel E. Mattox, George L. Wang, Chao Cheng

Generating regulon profiles

The mutual information for a lncRNA and its associated genes was computed using ARACNe-AP. Each mutual information value across all the genes interacting with the lncRNA was divided by the maximum mutual information. The values were then assigned a positive or negative sign based on the correlation coefficient (ρ_i) between the lncRNA's expression and the interacting gene's expression across samples to generate the final weight r_j.

$$1)\, r_i = sgn(\rho_i) * \frac{MI_i}{\max(MI)}$$

The profile was further split into an upregulated and a downregulated profile where:

$$2)\, r_i^{up} = \begin{cases} r_i, & if\ r_i > 0 \\ 0, & \text{otherwise} \end{cases}$$

$$3)\, r_i^{down} = \begin{cases} |r_i|, & if\ r_i < 0 \\ 0, & \text{otherwise} \end{cases}$$

Computing inferred expression using regulon profiles using BASE

A patient's gene expression profile is sorted in decreasing order to obtain **g.** The vector $\mathbf{r}^{\mathbf{up/down}}$ is the upregulated or downregulated regulon weight profile where the weights correspond to a lncRNA-gene edge. The weights are re-ordered so that gene labels of **r** and **g** match.

First, BASE moves down the sorted patient's gene expression profile **g** and calculates a foreground f(i) and background b(i) functions for both the upregulated and downregulated regulon weight profiles of the lncRNA.

$$1)\, f(i)^{up/down} = \frac{\sum_{j=1}^{i} |g_j r_j^{up/down}|}{\sum_{j=1}^{n} |g_j r_j^{up/down}|}, 1 \le i \le n$$

$$2)\, b(i)^{up/down} = \frac{\sum_{j=1}^{i} |g_j (1-r_j^{up/down})|}{\sum_{j=1}^{n} |g_j (1-r_j^{up/down})|}, 1 \le i \le n$$

Second, we calculate the pre-iExpr for upregulated and downregulated profiles and take their normalized difference to obtain the final iExpr for the lncRNA.

$$3a)\ pre\text{-}iExpr^{up} = f(i_{max})^{up} - b(i_{max})^{down}, where\ i_{max} = argmax(|f(i)^{up} - b(i)^{down}|)$$

$$3b)\ pre\text{-}iExpr^{down} = f(i_{max})^{up} - b(i_{max})^{down}, where\ i_{max} = argmax(\left| f(i)^{up} - b(i)^{down} \right|)$$

$$4)\ iExpr = \frac{pre\text{-}iExpr^{up}}{mean(\boldsymbol{n^{up}})} - \frac{pre\text{-}iExpr^{down}}{mean(\boldsymbol{n^{down}})}$$

pre-iExpr$^{up/down}$ is analogous to the D-statistic from the Kolmogorov-Smirnov test which compares the empirical distribution functions of two populations to determine if they are derived from the same distribution. $\boldsymbol{n^{up}}$ is a vector of null values generated by permuting the patient gene expression profile 500 times and re-calculating pre-iExpr for the upregulated profile. $\boldsymbol{n^{down}}$ is a vector of null values generated by permuting the patient gene expression profile 500 times and re-calculating pre-iExpr for the downregulated profile. These procedures were derived from the original BASE algorithm to fit this task.

Calculating meta z-scores in PRECOG

For each lncRNA, we fit a Cox proportional hazards model to its inferred expression in each dataset within a cancer type:

$$1)\ h(t|iExpr) = h_0(t)e^{\beta * iExpr}$$

We then extract the z-scores from each model that was fit to each dataset separately:

$$2)\ Z = (\hat{\beta})/se(\hat{\beta})$$

To combine the z-scores across the datasets within the cancer type, we implemented Stouffer's Z-score method:

$$3)\ meta\ z\text{-}score = \frac{\sum_{i=1}^{k} w_i Z_i}{\sqrt{\sum_{i=1}^{k} w_i^2}}, \text{where } w_i\ is \text{ the sample size of the } ith \text{ dataset}$$
